# Current Trends in the Treatment of Pediatric Hydrocephalus: A Narrative Review Centered on the Indications, Safety, Efficacy, and Long-Term Outcomes of Available Treatment Modalities

**DOI:** 10.3390/children11111334

**Published:** 2024-10-31

**Authors:** Dimitrios Panagopoulos, Georgios Stranjalis, Maro Gavra, Efstathios Boviatsis, Stefanos Korfias, Ploutarchos Karydakis

**Affiliations:** 1 Neurosurgical Department, Pediatric Hospital of Athens, ‘Agia Sophia’, 45701 Athens, Greece; 2 1st University Neurosurgical Department, Medical School, ‘Evangelismos’ Hospital, University of Athens, 10676 Athens, Greece; stranjal@otenet.gr (G.S.); skorfias@med.uoa.gr (S.K.); 3 Radiology Department, Pediatric Hospital of Athens, ‘Agia Sophia’, 10676 Athens, Greece; mmgavra@yahoo.com; 4 2nd University Neurosurgical Department, Medical School, ‘Attikon’ Hospital, University of Athens, 10676 Athens, Greece; eboviatsis@gmail.com; 5 Neurosurgical Department, General Hospital of Athens ‘Gennimatas’, 10676 Athens, Greece; karydakispl@gmail.com

**Keywords:** ventriculo-peritoneal shunt, ventriculo-atrial shunt, central catheter obstruction, infection, over-drainage, shunt independence

## Abstract

The pathophysiologic substrate of pediatric hydrocephalus has not been thoroughly elucidated. Valve-based shunt systems have constituted the main therapeutic option since the late 1950s. The initially used systems were concerning the ventricular system and the atrium. In the 1970s, VA shunts were not the main stay of treatment as the preferred option for the terminal end of the drainage system was the peritoneum. Our review analyzes these valve types and attempts a comparison, based on their functional characteristics. Nowadays, the only available surgical alternative for the treatment of hydrocephalus is ETV. This technique is associated with lower infection rates as well as, on average, a lower re-operation rate. Another term that deserves special mention is related to the outcome of ETV in children who had a medical history of previously incorporated shunts and who were subsequently suffering from shunt malfunction. Well-recognized predictive factors associated with secondary ETV failure include age, early onset of hydrocephalus, and prematurity. Although several attempts have been made in order to establish the optimum surgical treatment management in the different subgroups of patientswho are suffering from shunt dysfunction, there is no universal agreement. Therefore, this review attempts to identify the specific subpopulations of patients in whom the insertion of a drainage system as the preferred treatment modality is associated with an optimum long-term prognosis, compared to ETV, and vice versa. The objective of our study is to analyze the safety, efficacy, and outcomes of drainage devices and ETV in pediatric hydrocephalus patients.

## 1. Introduction

### 1.1. Overview, Definition, and Classification

Dandy had first described hydrocephalus as communicating and non-communicating (obstructive) in early 1913 and, after that, many more classifications have been suggested. In pediatrics, three main subdivisions are encountered: obstructive, communicating, and hypersecretory; the latter is mainly attributed to the over-production of CSF, mainly in tumors of the choroid plexus of the temporal horn of the lateral ventricles (papilloma-carcinoma). Congenital or developmental hydrocephalus is usually clinically evident at birth and is often considered a manifestation of a genetic syndrome or a manifestation or secondary occurrence due to spinal dysraphism.

In children, this condition predominantly manifests with a pathological elevation in intracranial pressure. In a limited subset of cases where this is not applicable, it is hypothesized that pressure remains within normal limits or low due to a compensatory mechanism that occurs elsewhere. This may interfere either with, and at the expense of, the brain parenchyma or by a mechanism that involves the expansion of the skull. In a minority of cases, both of the aforementioned mechanisms are interrelated [1,2]. The CSF normally flows from the lateral ventricles via the foramina of Monro, conducting into the third ventricle. In continuation, the bulk of CSF flows through the aqueduct of Sylvius (cerebral aqueduct), directed towards the fourth ventricle. After that, it exits the brain compartment and enters the spinal subarachnoid space. This is achieved through the foramina of Luschka and Magendie. Communicating hydrocephalus arises when the flow of CSF (within normal volumetric values) is unimpeded and this is caused by an inability to absorb CSF via the normal drainage pathways or, rarely, pathological accumulation of CSF due to over-production [3].

Although the diagnostic and differentiating criteria that are commonly utilized, such as “communicating” versus “noncommunicating”—in accordance with other classification schemes—are used, this is not always enough to delineate all subtypes of hydrocephalus. In clinical practice, we have to manage a wide spectrum of relevant conditions, among patients with hydrocephalus, which share in common a wide spectrum of clinical heterogeneity. In this review, we consider hydrocephalus primary (syndromic and/or idiopathic) or secondary to a wide variety of pathologic conditions.

Primary hydrocephalus may be driven by a range of genetic factors that have an effect on fetal development. Under this term, we refer to several entities, although they are not restricted to them, such as neural tube defects, arachnoid cysts, Dandy–Walker syndrome, and Chiari malformation [3]. Notably, a remarkable percentage of primary hydrocephalus cases in the pediatric neurosurgical practice are collectively included under the term idiopathic.

More precisely, our bibliographic review revealed that the term idiopathic hydrocephalus essentially entails two pathological entities. In more detail, one of them is named aqueductal stenosis, which constitutes a well-known anatomic substrate of obstructive hydrocephalus. This mainly refers to the pediatric population and can be managed efficiently with endoscopic third ventriculostomy. Preoperative magnetic resonance imaging is, in most cases, capable of undermining the pathophysiologic substrate of any particular aqueduct stenosis. There are several reported cases that mention follow-up evidence after a successful endoscopic third ventriculostomy. That is, a scheduled magnetic resonance brain imaging dictated the presence of a tectal plate glioma as the offending substrate of occlusion of the aqueduct. This could be attributed to a previously unrecognized phenomenon whereby concealed tectal gliomas that have clinically been detected due to hydrocephalus are only revealed after surgical management of the obstacle to the normal CSF flow and relief of the pressure-normalization of the dimensions of the ventricular system.

Another pathology that is included under this term is the entity of idiopathic external (benign) hydrocephalus in an infant. This is a pathological entity in which the head circumference is rapidly and excessively increased, thus exceeding significantly and persistently the values depicted by the Nellhaus curve. This figure is accompanied by a radiologically evident increase in the dimensions, mainly in the frontal, subarachnoid spaces. Although the term external hydrocephalus was introduced mainly to delineate a radiological feature, its widespread use was accompanied by misunderstanding and confusion among clinicians. As a result, we introduced the term benign enlargement of subarachnoid space when we were referring to infants as it better describes the benign natural history of this entity in appropriately selected infants.

Secondary hydrocephalus may occur mainly, but not solely, due to infection, bleeding, or trauma. In developed countries, PHH represents the most frequently encountered pathophysiologic substrate underlying secondary hydrocephalus in the pediatric population. PHH is most frequently associated with intraventricular hemorrhage due to premature rupture of ependymal vessels, which is universally accepted to be present in as much as 40% of premature newborns, whose gestational age was less than 37 weeks. It is thought that this is caused by the rupture of small, delicate vessels along the developing germinal matrix of the brain. These hemorrhages may occlude or scar the outlets of the ventricular system and/or obstruct the venous drainage pathways along the superior sagittal sinus (deep venous system) [3,4]. In developing countries, infections of the central nervous system, such as meningitis, constitute the most frequently encountered etiology of pediatric hydrocephalus, resulting in PIH due to inflammation of the ependymal lining and subventricular zone cells, as well as obstruction of the CSF drainage patterns at the pacchionian granulations, as well as an obliteration of CSF drainage or flow [3].

### 1.2. Hydrocephalus Treatment: The Evolution of Shunt Technology and the Development of Endoscopic Third Ventriculostomy

The first reported cases of hydrocephalus date back to Galen, Hippocrates, and the medieval Arabian physicians. Effective, durable treatment was unable to be registered and the first such successfully managed cases were recorded with Torkildsen in 1939 using an intracranial bypass system from the third ventricle to the cisterna magna to overcome an obstruction of the aqueduct of Sylvius [5,6]. The invention of a one-way valve drainage system did not occur until the 1950s [7,8]. Recently published studies have examined the evolution of shunt technology in the meantime [9,10]. During the 1950s and early 1960s, the value of the insertion of a drainage catheter within the ventricular system of patients suffering from hydrocephalus into the vascular system was verified [7,11]. Nulsen and Spitz placed the first valve, constituted by a spring and ball system. Later, this was replaced by a double-slit valve developed by Holter [11]. By 1969, at the Children’s Hospital of Philadelphia, 90% of shunt placements involved VA shunts with a Spitz-Holter valve. The major advantage of the VA shunt was that over-drainage was not a major concern in those patients with a closed fontanelle. Over-drainage with the VA shunt was rarely a concern since the atrial pressure was higher than the negative ICP in the erect position minus the gravitational column of CSF in the shunt tubing. The most serious disadvantages that were associated with the VA shunts are summarized as follows:

1. As the child grows, the peripheral catheter is gradually getting shorter and eventually needs to be elongated. Sometimes this is quite technically demanding due to clot formation around the tube in the lumen of the jugular vein;
2. If bacterial infection complicates our case and this involves the shunt system, the risk of development of septicemia is significantly increased.

Nevertheless, the invention of the VA shunt should be considered a major advancement in the treatment of hydrocephalus, being a life-saving procedure for many children. Apart from that, due to the potential side effects that were associated with its use, it became obvious that it could not be considered the therapeutic option of choice.

VPSs were rarely involved in the treatment of hydrocephalus in the 1960s, due to the high prevalence of obstruction of the distal catheters. In 1967, Ames [12] introduced a valve system whose peripheral catheter was ending at the peritoneal cavity. This shunt system demonstrated a favorable safety profile with minimal side effects and over the following few years, VP shunting replaced VA shunts, being considered the first option for drainage systems [13]. 

Although valve technology has progressed, there are several issues that need to be resolved. It is widely accepted that the most prevalent cause of shunt malfunction is obliteration of the central catheter [14,15,16]. The offending mechanism may be due to mechanical obstruction of the proximal catheter with choroid plexus or ventricular debris. Nevertheless, another contributory mechanism, potentially interrelated with over-drainage, is suspected. This predisposes individuals to ventricular catheter obliteration against the ventricular wall. Apart from the subgroup of children who present with non-functioning shunts and elevated ICP, there is another subgroup of children who present with symptoms attributable to intracranial hypotension, along with a working shunt. This is in accordance with the concept of over-drainage due to the inadequacy of the valvular system to overcome this complication [17,18,19]. The widespread availability of the imaging of the dimensions of the ventricular system in shunted children enhanced our knowledge about what is included under the term “SVS” or shunt inappropriate function, which is not coupled with ventricular enlargement [20]. Apart from that, a lot of referrals exist regarding children who are harboring well-functioning shunts, in association with small ventricular dimensions. A common complaint of children that are harboring a shunt refers to the frequent occurrence of headaches. This clinical observation, in conjunction with a relevant imaging study showing a ventricular system that is smaller than normal, altogether, was considered symptomatic over-drainage, attributed to the inserted system. The estimated incidence of either symptomatic low pressure or SVS varied from as low as 8–10% to as high as 40–50% [21]. The current hypothesis is that siphoning occurs when children transit from a supine to an upright position; this is capable of resulting in excess CSF drainage. In order to counteract the siphoning effect of currently available valvular mechanisms, antisiphon devices were invented to be inserted in line with the valves [22]. The beneficial results reported were not verified by all of the initially published series. More precisely, some publishments demonstrated a significant diminishment in the rate of proximal catheter obliteration, whereas others were unable to support any evidence of benefit [23]. When standard differential pressure valves were utilized, the rates of shunt failure remained largely unchanged and relatively high, with approximately 40% of shunts associated with malfunction within the first year of insertion [24]. Newer technical innovations have been introduced, referring to both the shunt and the ventricular catheter, in order to overcome side effects associated with either to over-drainage or the issue of shunt malfunction due to obliteration. These technical advancements involve the utilization of programmable valves with externally adjustable opening pressures, valves with antisiphon devices, gravity-assist devices, and flow-regulating valves [25]. Current valve technology includes the following:

1. Fixed differential pressure (DP) valves;
2. Over-drainage control devices (OCDs);
3. Adjustable DP valves;
4. Fixed DP valves with OCDs;
5. Adjustable DP valves with OCDs.

### 1.3. Lumboperitoneal Shunts

LPs have been utilized in the management of a wide spectrum of pathologies that are included under the term communicating hydrocephalus. Nevertheless, the most widely accepted indication is for the treatment of idiopathic intracranial hypertension as the lumbar subarachnoid space allows access for CSF diversion, even in the presence of a ventricular system that radiographically seems to be slit. These types of shunts have also been implicated with a lower infection rate, as well as with a lower prevalence of obliteration of the central catheter [26,27]. The initially introduced types of LP shunts mostly used slit valves, which are characterized by a simplified hardware design, which reduces the likelihood of failure. Nevertheless, a significant limitation of these valve types is that they are able to offer limited control regarding CSF flow, thus, they could be involved in cases of shunt over-drainage [28]. In a retrospective review of 143 pediatric patients harboring LP shunts, 70% of patients showed radiographic evidence of tonsillar herniation on follow-up imaging. This should be attributed to the resultant over-drainage of CSF. Nevertheless, only 4% of this subpopulation of patients ultimately necessitated any intervention [29]. These shunt systems share in common another inherent drawback, that is they do not offer the opportunity to clinically assess their functional status. This limitation stems from the absence of an access reservoir, which enables aspiration and measurement of the opening pressure. LP valves have been introduced to our therapeutic armamentarium in order to more effectively control CSF flow and a reservoir can be connected with them. The Integra horizontal-vertical valve incorporates two different valve mechanisms, a spring-actuated valve that controls a lower pressure range of drainage while the patient is horizontal and a gravity-actuated valve controlling higher-pressure drainage when the patient adopts an erect position [21]. When the literature data that are relevant to the cumulative rate of complication associated with LP shunts are analyzed, it seems that they are not enlightening. More precisely, there are several reports that mention an increased failure rate, thus necessitating shunt revision [30,31]. However, a recently published retrospective study demonstrated that the rates of shunt failure, number of failures, and overall complication rates were comparable to those associated with VP shunts [32].

### 1.4. Endoscopic Third Ventriculostomy

At present, endoscopic third ventriculostomy is the sole surgical alternative option to shunt placement for the management of hydrocephalus. Since 1910, multiple efforts have been undertaken to refine an endoscopic approach to the ventricular system. Hoffman [33] reported on a cohort of children with non-communicating hydrocephalus treated effectively with ETV and Cinalli [34] published his results based on the encouraging results of third ventriculostomy in patients necessitating shunt revision. He demonstrated that 76.6% of patients became shunt-independent with a mean follow-up of 7 years. Better lighting and visualization, which were made possible by technological improvement by the late 1990s, attracted increased interest from neurosurgeons. These technical innovations facilitated us to attempt to treat, with third ventriculostomy, not only pathologies related to non-communicating hydrocephalus but also patients suffering from communicating hydrocephalus [35,36]. The outcomes were highly promising [37,38], the only exception being infants and children under 1 year, especially those under 6 months [39]. In all groups, the failure rates associated with ETV were higher than their counterparts, associated with shunts, when the evaluation period was restricted in the first few months. Nevertheless, when the investigation period was extended over the next few years, ETVs were associated with fewer revisions and, accordingly, demonstrated a lower incidence of failure [40,41,42]. After an 8-year follow-up period, the revision rate for ETV (which included a revision of ETV or the insertion of a shunt) was 49%, with failures occurring late, in the time range of 5–8 years [43]. A scale was developed in order to be able to predict which patients would be most likely to benefit in the long term from ETV [44] called the ETVSS. The ETVSS seems to be a valuable predictor of successful outcomes [45,46] and may even undermine the success rate of ETV [42]. A modification of this score has been introduced [47]. Nowadays, more than 50% of children suffering from hydrocephalus should be considered for ETV as the optimum treatment option. A recently introduced technique incorporates the performance, in addition to ETV, of choroid plexus cauterization in infants. However, the relative benefits of this are still under investigation [48,49,50]. The rationale for their use primarily hinges on the observation that the pathological substrate of the hydrocephalus, e.g., post-infectious, may be an important determining factor that can define the potential (expected) benefit from choroid plexus coagulation [51]. 

## 2. Materials and Methods

The vast majority of recently published review articles centered on hydrocephalus have concentrated on particular parameters of its pathophysiology. In accordance with that, we would like to mention to our readers the following topics that most review papers deal with: CSF and the ventricular system [52,53], hemorrhagic hydrocephalus [4,54], congenital hydrocephalus [4,55,56,57], and idiopathic hydrocephalus [58,59,60]. The purpose of this review article is to thoroughly, and in total, document the whole spectrum of the pathophysiology of hydrocephalus as an entity, to review the currently available management protocols, and compare the available treatment modalities in terms of relative indications according to the underlying pathology, safety, and long-term efficacy, as well as future potential considerations.

Relevant data were collected utilizing PubMed, Google Scholar, BioRxiv, and MedRxiv search terms: hydrocephalus, CSF, ventricles, ventricular system, and intracranial pressure. We did not adopt exclusion criteria, thus our search protocol gathered articles of all subcategories (reviews, original articles, clinical trials, etc.). Finally, no restriction criteria were inserted, referring to the year of article publication. For the purposes of ensuring wording accuracy, we would like to mention that the first VP shunt system was introduced by Holter and Spitz in 1956. This means that, for practical reasons, this date constitutes the earliest publication year limit of our literature search. These articles were reviewed via title and abstract in order to make certain that their main point of interest was related to hydrocephalus that is to control ICP along with ventricle size. The basic tenet of the authors of this review article is to provide historical, as well as background and updated, information about the treatment armamentarium for hydrocephalus; a detailed analysis of the current categorization of hydrocephalus based on the pathophysiology of this entity; and a presentation of the currently available established options, focused to the treatment of hydrocephalus.

## 3. Discussion

### 3.1. Treatment Paradigm Standards of Surgical Intervention

When we are dealing with patients who are suffering from hydrocephalus, there are several options in our therapeutic armamentarium regarding the intervention procedure. Our final treatment decision depends on a wide spectrum of variables, including patient weight, severity of symptoms, and clinical recordings. All of them are fairly well analyzed by a systematic bibliographic review and literature-based guidelines, which are included in recently published manuscripts [61,62]. In general, they are subdivided into transient surgical interventions, as well as permanent surgical treatments. 

Temporary surgical measures for hydrocephalus include the insertion of an EVD or the development of a VSGS. The choice of incorporating NEL into our treatment plan, aiming to suction or dilute the intraventricular debris, resulting from infection and hemorrhage, is currently under investigation using the international multicenter “TROPHY” registry [63]. 

Permanent management strategies for hydrocephalus involve two basic options, which are based on a completely different mechanism of action. One of them attempts to overcome an obstruction obstacle, which was due to an anatomical dome, to the flow of CSF (i.e., tumor of the third ventricle) using neuroendoscopy (e.g., ETV). The other main subcategory of therapeutic interventions incorporates the insertion of a shunt in order to facilitate CSF diversion from the ventricular system to a body cavity, which can effectively absorb the excess fluid. The most common options include the peritoneum, atrium, or pleural cavity. However, it is well known among pediatric neurosurgeons that permanent CSF diversion procedures are generally prone to a significant rate of failure and, frequently, a re-implantation of the shunt system is required. 

Currently, the most commonly used shunt type is the VPS, which is thought to be our first-choice approach for the greater percentage of patients. Nevertheless, while the peritoneal cavity is the most frequently selected location for the drainage of CSF, VA and VPlS are currently considered acceptable alternative distal targets in cases where the abdomen is deemed to be inappropriate. 

There are different types of shunt systems available, namely, pressure-modifiable vs. pressure-non-modifiable, and valve systems that include an antisiphon device, which could be programmable or non-programmable.

As is well known, one of the most common and frequently devastating complications of the shunt systems is associated with chronic shunt over-drainage and, secondarily, there is the development of the entity of slit ventricle syndrome. It is common practice to upgrade the differential opening pressure of pressure-modifiable valves in cases of the establishment of these side effects. Although prospective, double-blinded multicentric reviews are lacking, there is a widespread agreement that this up-regulation of the opening pressure of the valve system could be considered the first step of our therapeutic armamentarium regarding the management of these entities. However, the detailed description of this entity is out of the scope of our review and it will not be analyzed in more detail.

Neuroendoscopy is a surgical technique that can be used as an alternative option to shunting therapy in a subcategory of patients who share in common several prerequisites, as well as specific indications. More precisely, they involve a subpopulation of patients who present with obstructive hydrocephalus, regardless of its underlying pathophysiology and etiology. It involves the placement of an endoscope into the ventricular system in order to address any underlying primary pathology or to provide an alternative pathway for the bulk flow of CSF. Walter Dandy is assigned as the one who first attempted to utilize the neuroendoscope in order to address the excess flow of CSF and popularized ETV in the early 1900s [64]. This technique was effective in resolving obstructive hydrocephalus at the level of the third ventricle by the development of a septostomy, thus creating communication between the floor of the third ventricle (after perforation of the premammillary membrane) and the subarachnoid space (that is chiasmatic, interpeduncular, and prepontine cisterns). Initially, ETV-related morbidity and mortality was unacceptably high. This should be attributed to severe inherent limitations of the visualization capacity, with early patient series demonstrating mortality in the range of 75% [64].

ETV underwent a resurgence in popularity from the beginning of the 21st century, mainly due to the work of Dr. Benjamin Warf, which was based on patients from Sub-Saharan Africa. The fact that they shared in common was the restricted access to shunt devices [65,66]. His patient cohort, based on the utilization of ETV, has shown satisfactory management of hydrocephalus, which fluctuated in the range of approximately 80% of his patients. ETV is sometimes followed by CPC. The theoretical basis under this manipulation was to decrease the amount of CSF produced by the choroid plexus of the lateral ventricles. There are a number of risk factors that are associated with an increased likelihood of failure of the ETV. These include younger age, hydrocephalus of non-obstructive origin, and pre-existence of a drainage system. The combined interaction of all these factors constitutes the main reason for forming the ETVSS, which is utilized as a tool in order to predict whether it is reasonable to attempt an ETV in patients harboring specific characteristics [44].

It is a common concept that several valuable improvements are established in our technical and theoretical perspective in the context of CSF diversion as a treatment option for hydrocephalus. Nevertheless, infection and treatment failure due to shunt obstruction, complicate the treatment strategy of these at-risk patients. Especially in children, the estimated prevalence of shunt failure has significantly increased, with a frequency of up to 50% in the first two years [67,68,69]. The most frequently recorded causes of shunt failure are categorized under the general terms ‘mechanical reasons’ (catheter breakage or disconnection), malposition (central end of the catheter not in the ventricular cavity, peripheral end out of the peritoneal cavity), or obstruction (choroid plexus ingrowth or debris, epithelialization). By definition, each case of shunt malfunction necessitates a minimum of one re-operation, with any further attempt at shunt repair being associated with an increased risk of morbidity and mortality [70]. ETV with or without CPC is considered to be able to alter the clinical evolution of these patients as they could impair the long-term management of hydrocephalus, depending on its underlying etiology. Based on that, cases associated with long-term failures that necessitate revision are common. The Kaplan–Meier estimate curves for patients with a 60–70% ETVSS cross at 6 months following the index procedure. 

### 3.2. Comparison of Endoscopic Third Ventriculostomy and Shunt Placement in the Pediatric Population

Hydrocephalus in the pediatric population constitutes one of the most frequently encountered diagnoses, registered at admission in the pediatric neurosurgical units [71,72]. Currently, a VPS is considered the standard of treatment, although a lot of advancements have been achieved and several new CSF diversion approaches have been introduced in the management of hydrocephalus. A very promising approach that has been included in our range of therapeutic options for the management of this entity includes the ETV [73,74]. 

Nevertheless, the VPS is implicated with a wide spectrum of adverse effects, including shunt failure, regardless of the underlying cause; infections; and unpredictable long-term motor and cognitive-neurological status [75]. A lot of clinical trials have attempted to compare and evaluate the present shunt success and relevant failure rates in comparison with those of the previous decades; however, the existing data in the literature are inconsistent and, thus, unable to extract a definite conclusion [76,77].

ETV has been established as an alternate procedure for the management of CSF circulation disorders, most commonly preferred in cases of hydrocephalus that are non-communicating in terms of their underlying cause [78,79]; apart from that, it has been proposed that ETV might also be useful for a specific subgroup of pediatric patients who are suffering from communicating hydrocephalus [34]. A recent trend includes the addition of CPC during ETV. Preliminary results have been published, supporting that the efficacy of the endoscopic approach is enhanced [66,80]. This new therapeutic trend is scientifically supported by the growing number of studies that attempt to evaluate and compare the relative effectiveness of VPS and ETV [81,82].

One of the objectives of our narrative review is to systematically evaluate the literature and analyze the relative safety and efficacy of valve-based drainage systems and ETV in cases of pediatric hydrocephalus. Most of the involved studies share in common several intrinsic limitations that weaken the validity of their results. Some of these include the finding that in the overwhelming majority of patients, the etiology of hydrocephalus is mixed, as well as the fact that geographical location and technical differentiations in the performance of ETV influence our patient’s ultimate outcome. However, the aforementioned studies have tried to investigate the relevant effect of all of these parameters by conducting subgroup analyses.

The extracted conclusions verified that patients who underwent ETV shared in common a statistically significant decrease in the incidence of any infections that are related to the offending procedure. This was reaffirmed in the analysis of the subgroup of cases that included only patients suffering from obstructive hydrocephalus. No differentiation regarding the terms of repeat operations, mortality, and CSF leaking was identified in the original pooled analyses that were based on the comparison of these two groups [71].

Despite the fact that the VPS method has been performed as an operation for several decades, a lot of complications continue to be intimately related to it, including procedure-related infections; CSF leaks; an increased occlusion rate, along with a referral of several re-operations; and even mortality [72]. When ETV was considered to be a promising alternative to shunt procedures, the initial indication for its use was strictly diminished to cases of hydrocephalus associated with stenosis of the aqueduct of Sylvius. Nevertheless, the spectrum of indications was gradually expanding. This fact was also evident when we were recording the indications for the performance of an ETV. All our data were included in the studies that were incorporated as part of a relevant meta-analysis based on obstructive and communicating hydrocephalus cases [74,83]. ETV and shunt procedures can be alternatively utilized for cases with pathologies that do not rule out any one of them. Nevertheless, there is always a number of variables, based on individual patient characteristics (i.e., previous CNS infection, number of previous attempts at shunt revision, patient age), which could predict the anticipated success rate of the procedure and influence ultimate patient selection. 

Infections are generally considered to be one of the most common and devastating adverse effects in procedures involving CSF diversion [81]. Although several attempts have been performed by organizations, such as the Hydrocephalus Clinical Research Network, shunt infection constitutes a major issue, adding significantly to the overall morbidity and mortality [84]. The previously reported meta-analysis seemed to verify that ETV is inherently related to a statistically significant reduced prevalence of infection, associated with the operation itself, compared to shunt insertion, during the post-operative period of clinical surveillance. Nevertheless, there was no individual study, focused on this comparison, that was capable of supporting on its own these statistically significant differences between ETV and shunting. It could be supposed that these studies were inherently unable to individually prove this difference; nevertheless, the utilization of meta-analysis was able to substantially enhance the statistical power and, therefore, statistical significance was detected. 

When we encounter patient cases where the primary CSF diversion procedure fails, a re-operation is commonly needed. This could involve a repeat of the first procedure or the application of another technique [74]. According to the studies that are included in the reported analysis [71], the relevant details that would refer to the technical aspect of the repeat operation were lacking. This inconsistently present information is a fact that constitutes an inherent limitation. The attempted pooled analysis was not capable of verifying any significant differences between ETV and drainage procedures when re-operation was the term that the comparison was based upon. 

The aforementioned study suggested that, when all cases of mortality of any cause and rates of CSF leaking were compared between the two main treatment modalities, the results from the ETV and shunt groups did not show any significant difference. These conclusions are in alignment with the individual RCT published by Kulkarni et al. [41], who were not able to document any statistically significant differences based on the comparison of the data from the included study groups, in terms of mortality, regardless of the underlying cause. CSF leaking was not mentioned in this RCT but it was in accordance with all relevant studies that were included in this RCT and contributed to this outcome. Future RCTs are mandatory in order to enhance the validity of the results of these published series. In particular, future studies would be able to yield more valuable conclusions if they could be based upon widely accepted definitions and on methodologically standardized outcome measurements, centered on the definition criteria of success and failure of ETV or shunting. Moreover, other prerequisites include the ability to compare only subgroups that share in common a uniform age, a common etiology for the hydrocephalus, as well as a follow-up period that is predefined from the beginning.

### 3.3. Comparison of Endoscopic Third Ventriculostomy and Ventriculo-Peritoneal Shunt Placement in Infants and Children in Terms of Safety and Efficacy

We reviewed studies comparing ETV and shunts in pediatric populations, with the goal of providing consultation in the selection of the optimum treatment strategy [85]. According to our cumulative data, we should suggest that there are no statistically significant verified differences in terms of success and failure rates within 1 year after surgical intervention between ETV and shunting. Therefore, based on that evidence, at present, there is no surgical strategy that is justified to be considered as significantly advantageous. Cheng et al., in a recent meta-analysis, reported that ETV and VPSs can be used as alternating therapeutic options, without any compromise of the final patient outcome, for the management of obstructive hydrocephalus in heterogeneous populations of adults and children. Nevertheless, ETV is inherently accompanied by decreased surgery time, postoperative complications, and re-operation rates. On the other hand, there are several authors who mentioned that ETV should be considered a more effective treatment modality in the subgroup of pediatric patients that includes those who are older than 2 years of age [86,87]. On the contrary, others demonstrate cumulative data that the underlying pathological substrate is a more important determining factor than age [88]. Rasul et al. stated that although both ETV and shunting techniques are accompanied by an increased rate of failure, there are some remarks suggesting another concept. According to them, ETV may be inherently related to an improved long-term rate of success [87,89]. No significant difference was established, when ETV and shunting were compared, in terms of both success and failure rates at one-year follow-ups. Koch et al., in a relatively recently published review, showed that the failure rate that accompanies ETV increased, up to 68.8%, within a median of 38 days [90]. On the contrary, other recently published series report a failure rate in the range of 26.5% [91]. In a recent systematic review, Bouras et al. mentioned that the rate of complications that accompany an ETV procedure was in the range of 8.5%, whereas the mortality risk was 0.28% [92]. They attributed that improvement mainly to advanced technology, as well as to improved surgical skills. On the contrary, shunt-based treatment protocols, although they were combined with a low risk of mortality (0.1%) [76], are constantly accompanied by high failure rates (31.3% for the first year and 4.5% per year thereafter), with no significant advancements over the past several years. Except for the efficacy and failure rates of any individual treatment, a constellation of other parameters should be revealed during the selection of the best management option. Such variables include, although they are not restricted to, relevant patient anatomy and baseline clinical features, age of the patient, pathophysiology of hydrocephalus, factors that are intimately related to the operating surgeon, and equipment availability. 

### 3.4. Shunt Independence and the Role of Endoscopic Third Ventriculostomy

There is only a restricted bulk of evidence dedicated to the issue of shunt independence, although this is a permanent, and of paramount significance, concern for patients to whom an internal drainage system is implanted. Moreover, treating neurosurgeons play a pivotal role, as they are dealing with potential shunt-related complications [34,93,94,95,96,97,98,99,100]. Another issue that further complicates this discussion and necessitates further determination is centered on the determination of a generally accepted definition of shunt independence, the most effective method to attain it, and what the real prevalence of that outcome is. Based on the fact that there is a lack of relevant prospective studies and also on the consideration that there is only a minority of patients who present with spontaneous independence [97], shunt removal is frequently elective. Moreover, this maneuver is frequently related to our efforts to calculate the estimated success rate of secondary ETV [101] and is usually performed in patients with a medical history of frequent shunt dysfunctions and revision surgeries [34]. The estimated rate of accomplishment of shunt independence is currently in the range of 3 to 9% of pediatric patients suffering from hydrocephalus [98,99].

Shunt independence has been the ultimate tenet for neurosurgeons since shunt surgery became a common clinical practice. According to several anecdotal reports, it has been intimately related to their efforts to handle shunt-related complications. These are more frequently associated with shunt over-drainage and their efforts to prevent or even treat this adverse effect of shunt devices. In this context, several technical innovations have been adopted in order to facilitate independence (intermittent cranial compression with ICP monitoring, “on-off” types of shunts, subtemporal craniectomy) [98]. Several meetings discussed the issue in the late 1980s and early 1990s (Shunts and Problems in Shunts Symposium, Marseille, June 1980; Consensus Conference: Hydrocephalus ‘92, Assisi, Italy). Several proposals have been submitted in order to better discriminate the terms shunt independence and compensated or arrested hydrocephalus. Based on that, initial efforts to define the concept of shunt dependency were recorded, albeit with equivocal conclusions. The prevailing view among the scientists at that time was that no individual test is reliable on its own and the safety of intentional shunt removal remains uncertain. According to the policy that was adopted recently by a large pediatric department [93], secondary ETV is more often proposed for individuals suffering from obstructive hydrocephalus of primary or acquired origin [102]. These patients usually share in common a medical history that is complicated with recurrent episodes of shunt malfunction or a medical history of complex surgical procedures, attempting to handle the issue of shunt malfunction. The whole intervention is usually accomplished as part of a treatment algorithm, usually via prior emergent externalization of the shunt or EVD surgery.

### 3.5. Endoscopic Third Ventriculostomy and Infant Patient Population

There are ample, albeit contradictory, referrals from pediatric patients centered on which individuals are most likely to benefit from ETV [103]. These controversial results constitute the basis for a debate that mainly centers on the success rates of ETV, the significance of age as an isolated factor, the aetiology of hydrocephalus, or both. Some neurosurgeons have advocated for ETV as the management modality of choice for the obstructive subtype of hydrocephalus, which is attributed to primary aqueductal stenosis and other closely related pathological situations [104]. More precisely, according to the previously mentioned study, ETV was selected as the most appropriate surgical option in 96.5% of individuals who were incorporated in that study. Nevertheless, this study was unable to verify if patients who were less than 1 year of age shared in common a higher risk of ETV failure when a comparison was made with older participants. The authors mentioned that when all included studies were taken into consideration, the success rate was 51.6%. When individual published reports were taken into consideration, the reported success rates were ranging from 0% [105] to as high as 83% [106]. There were published studies that recorded lower success rates when they were dealing with younger patients [107,108,109]. However, the aforementioned results were not always supported by a level of statistical significance [79,106,110]. One report concluded that the underlying pathology and not the patient’s age was the main determinant; that is the factor that was influencing the outcomes of ETV. More precisely, patients with an underlying diagnosis of congenital aqueduct stenosis were inherently associated with a better long-term outcome compared with their counterparts associated with other offending pathologies [111]. Moreover, an outcome that was dependent simultaneously on the patient’s age and aetiology of hydrocephalus was reported by four studies [91,112,113,114]. One publishment stated that patients with aqueductal stenosis who belong to a younger age group, as well as those who are suffering from Chiari malformation, had statistically significantly worse outcomes [112]. The other relevant studies concluded that older infants with stenosis of the aqueduct were intimately related to improved outcomes [91,113,114]. 

Recently, a meta-analysis was published, centered on the role of ETV in patients with a manifested shunt malfunction who belong to the pediatric population [114]. In conclusion, it manifests that when secondary ETV is selected as the treatment modality in this cohort of patients, it should be regarded as an acceptable option, accompanied by relatively good success rates, along with low complication rates. Based on those remarks, it could be justified as worth considering for cases that are complicated by shunt malfunction.

### 3.6. The Role of Neuroendoscopy in the Management of Post-Infection Hydrocephalus

The management of multiloculated hydrocephalus remains an intractable problem, mainly due to our inability to simultaneously and effectively drain compartments that are in isolation from the remaining ventricular system [115]. The ultimate target lies in the creation of a single, freely communicating cavity that could be effectively drained by one ventricular catheter after establishing a communication between the different entrapped compartments [116,117,118,119,120,121,122]. The more traditional treatment algorithm includes the insertion of multiple, separate ventricular catheters, which could be able to effectively drain non-communicating compartments. The main drawback of this approach is that, by increasing the overall complexity of the shunt system, it is far from desirable as it multiplicates the risk of complications, mainly that of central catheter obstruction [116,120,122,123,124]. Wide fenestration of the membranes separating the individual ventricular compartments appears to be the procedure that is more advantageous and more akin to normal CSF circulation. The sole means to achieve this is through open microsurgery or endoscopic surgery [116,117]. Endoscopic fenestration has inherently been correlated with reduced blood loss and decreased operative time, as well as with lower morbidity and a reduced time period of hospital recovery [116,120]. An alternative option, as well as complementary, includes the placement of proximal catheters via the aid of endoscopic guidance. This may also be associated with a decreased risk of shunt malposition [125]. Therefore, endoscopic fenestration nowadays consists of the optimum management option for multiloculated hydrocephalus and this is more evident in infants. Apart from that, open fenestration has currently been our ultimate solution, reserved for severe or refractory cases [116,118,120,122].

### 3.7. Posthemorrhagic Hydrocephalus in Premature Infants and Available Treatment Modalities

We have performed bibliographic research in an attempt to determine the current, if any, recommendation guidelines concerning the optimum time frame of shunt insertion in premature infants. Based on our literature review, there are inadequate data, that is, they are unable to definitely document a specific weight or a peculiar CSF value or to determine the optimum time point of shunt insertion in premature infants with PHH [126]. On the contrary, clinical judgment is always required in such cases. Based on the guidelines that were extracted from an extended literature review, the current strength of recommendation is Level III, that is, not absolute clinical certainty. Another relevant issue is centered on the current recommendation concerning the utilization of ETV. According to the previously reported reference, there is not much evidence able to justify the utilization of ETV in premature infants suffering from posthemorrhagic hydrocephalus. The stage of recommendation is Level III, meaning no verified clinical certainty.

### 3.8. The Entity of the Isolated Fourth Ventricle and Available Treatment Options: Relative Advantages and Disadvantages

A TFV is a relatively uncommon, as well as critical and difficult-to-manage, pathological entity, which usually refers to patients who have suffered from an intraventricular hemorrhage, inflammation or infection, and tumor resection that involved the fourth ventricle, along with ventricular shunt placement. It is usually coming to clinical attention by the delayed onset of its clinical signs, following a period of relative neurological amelioration [126,127]. The pathophysiological mechanism that is implicated in cases of a TFV is intimately related to the arachnoidal blockage of the inlets and outlets of the fourth ventricle, the aqueduct of Sylvius, and the foramina of Magendie and Luschka, respectively [128]. The available management strategies consist of the placement of a separate drainage system (central catheter within the fourth ventricle) and endoscopic and microsurgical fenestration. Nowadays, in the vast majority of cases, the selected treatment modality is the insertion of a shunt with a central catheter directed into the fourth ventricle [129]. Nevertheless, this option is intimately related to several complications, including infection, malposition, malfunction, and brain stem injury [130]. Nowadays, taking into consideration the widespread evolution of endoscopic techniques, a lot of published reports have presented individual cases of patients treated by endoscopic aqueductoplasty, either with or without stent placement [131]. The ultimate tenet of these techniques is to restore the communication between the TFV and the third ventricle or the subarachnoid space, thus circumventing the dependence from a separate fourth ventricular drainage system.

Endoscopy should be regarded as the most appropriate management strategy and this should be attributed to its significantly decreased revision rate and its tendency to be correlated with a significantly higher rate of clinical improvement, in comparison with shunt placement. Another viable and effective treatment option remains the use of an open microsurgical fenestration. This is further supported by the fact that it is accompanied by a similar clinical outcome and its revision rate is comparable to that of endoscopy. However, we should underline the fact that it is inextricably linked to a more invasive surgical approach and, thus, should not be considered a first-line treatment option. Shunt insertion could be managed as a rescue type procedure in the treatment of a TFV as it is frequently accompanied by serious complications and associated with an increased rate of failure, and subsequent revision, compared to endoscopy. Endoscopy could even be considered the most appropriate management option in the infantile population (<1 year old). This can be attributed to the fact that it is a minimally invasive procedure and there is no definitive evidence that can justify its inferiority when compared with other treatment options.

Hydrocephalus is the most frequently encountered illness, as well as being accompanied by devastating complications, affecting a fetus. The development of intrauterine ultrasonography, as well as MRI, accompanied with laboratory tests, has added a lot to our diagnostic armamentarium. Based on that, our review attempts to summarize the existing data referring to the possibility of the application of the aforementioned surgical techniques in the neonatal period. It seems that, in recent years, several studies in this area are present in the literature, both reviews and case reports.

The most common underlying causes of hydrocephalus that could potentially be treated in utero consist of aqueduct stenosis, intraventricular hemorrhage, Dandy–Walker malformation, and Chiari type II malformation. Some fetuses underwent the insertion of a ventriculo-amniotic shunt and others fulfilled the criteria for endoscopic third ventriculostomy. Apart from that, after birth, ventriculo-peritoneal shunting with low-pressure valves was the selected treatment option for a significant percentage of patients.

Consequently, there is a current trend that when chromosomopathies are lacking and when the gestational age is within the range of 24 and 32 weeks, deteriorating fetal hydrocephalus that is not attributed to an infectious etiology could have a beneficial clinical outcome if intraventricular decompression is performed during the intrauterine life. This could be a viable option for individual cases of isolated, evolutive, devastating hydrocephalus diagnosed before 32 gestational weeks, which may benefit from intrauterine neurosurgical manipulations [132,133,134,135].

**Figure 1:**
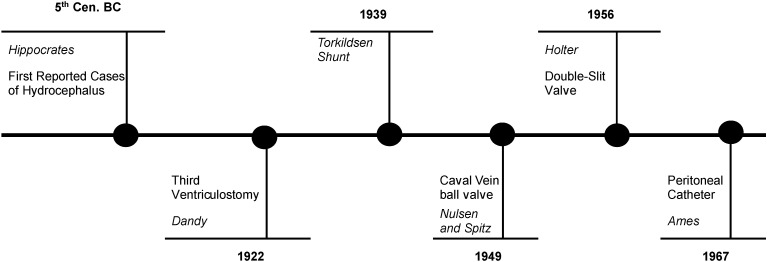
Schematic diagram representing the main historical evolutions in the diagnosis and management of hydrocephalus.

Figure 1, Schematic diagram representing the main historical evolutions in the diagnosis and management of hydrocephalus 

**Figure 2:**
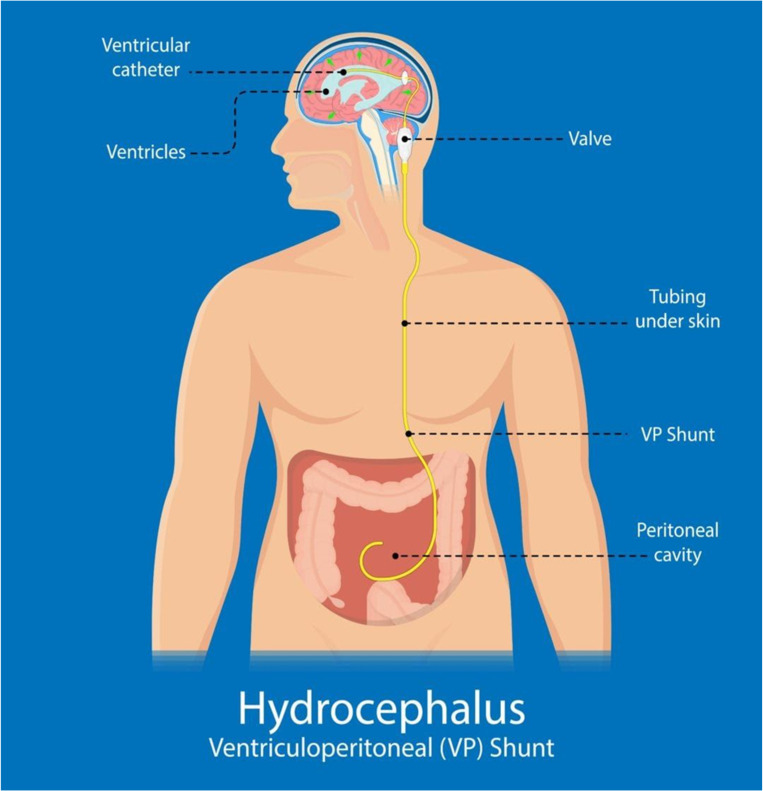
Schematic representation of a ventriculo-peritoneal shunt.

**Figure 3:**
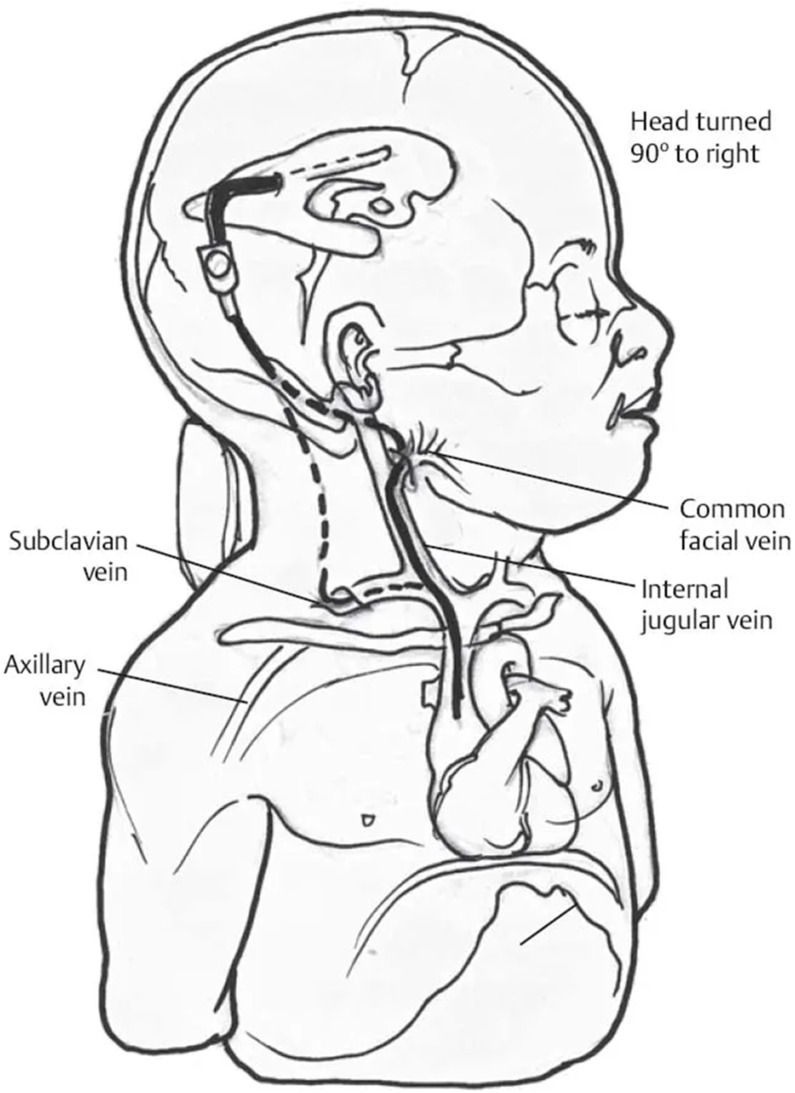
Schematic representation of a ventriculo-atrial shunt.

**Figure 4:**
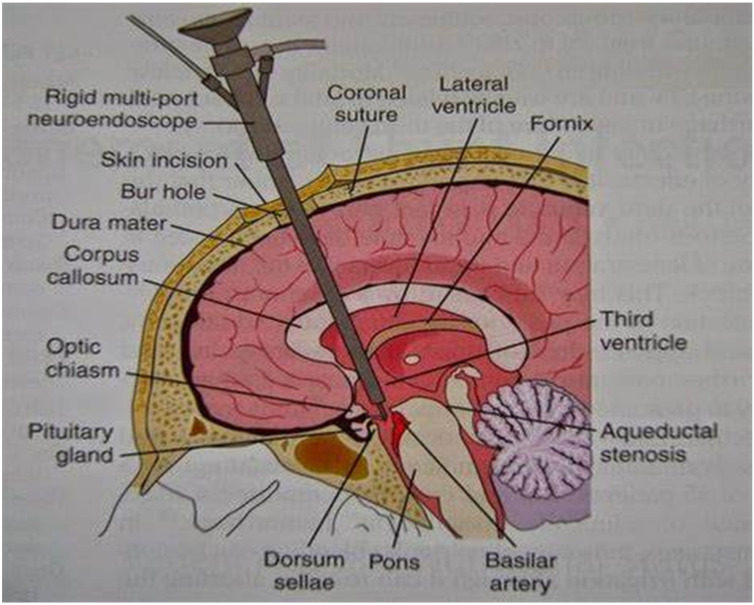
Content. Schematic representation of endoscopic third ventriculostomy from an anatomical point of view.

The following Figure 2, Figure 3 and Figure 4 attempt to diagrammatically illustrate the described techniques, VPS, VAS, and ETV, respectively.

## 4. Conclusions

ETV is generally related to a statistically significantly decreased risk of operation-related infection in comparison with shunt placement. Apart from that, overall mortality rates, CSF leaking, and re-operation rates seem to be within the same range when the two groups of patients are compared.

As far as we were able to know, based on the current literature data, ETV and shunts are associated with similar 1-year success, as well as failure, rates. Based on that, there is no justified data that could be able to recommend one procedure over the other. Definitively, there is a need for the execution of more randomized studies with age and etiology subgroup analysis. These are needed in order to identify the most effective option regarding the treatment modality that should be followed for all of the divergent groups of hydrocephalus patients.

Shunt independence following a subsequent ETV in cases of obstructive hydrocephalus, performed at the time of shunt malfunction, has significantly enhanced the rate of success and should be discussed in a selected, yet appropriate, subpopulation of patients. Intended removal of the shunt in cases of symptomatic shunt over-drainage constitutes a procedure that is accompanied by significant risks and should only be discussed in a particular subset of patients that fulfill specific and strict criteria. These criteria include close monitoring and the ability to obtain a long-term follow-up. There is compelling evidence that supports the concept that secondary ETV in pediatric patients is an alternative option, with encouraging success rates and low complication rates, and merits consideration in individuals suffering from shunt malfunction.

ETV has been proven to be a valuable tool in our therapeutic armamentarium in order to avoid shunt placement in selected cases of post-infective hydrocephalus. Moreover, there is cumulative evidence to support that this could be accompanied by a high success rate in selected cases of shunt malfunction, associated with infection or previous post-infective hydrocephalus.

ETV can be considered an effective and safe alternative for children experiencing shunt dysfunction, being able to offer shunt independence in cases that complete several indications and prerequisites: primary obstructive hydrocephalus with aqueductal stenosis and post-inflammatory hydrocephalus in children ≥36 months.

## Data Availability

The original contributions presented in this study are included in the article. Further inquiries can be directed to the corresponding author(s).

## Abbreviations

ETV	endoscopic third ventriculostomy
CSF	cerebrospinal fluid
PHH	post-hemorrhagic hydrocephalus
CPC	choroid plexus cauterization
RCT	randomized controlled trials
PHH	post-hemorrhagic hydrocephalus
IVH	intraventricular hemorrhage
PIH	post-infectious hydrocephalus
ICP	intra-cranial pressure
TFV	Trapped or isolated fourth ventricle
VA	shunt: ventriculo-atrial shunt
VPlS	ventriculo-pleural shunt
VSGS	ventriculo-subgaleal shunt
NEL	neuroendoscopic lavage
LPs	Lumboperitoneal shunts
SVS	slit ventricle syndrome
VPS	Ventriculo-peritoneal shunts
VPlS	Ventriculo-pleural shunts
EVD	external ventricular drain
ETVSS	ETV success score
